# The Dose-Response Relationship Between Body Mass Index and the Risk of Incident Stage ≥3 Chronic Kidney Disease in a General Japanese Population: The Ibaraki Prefectural Health Study (IPHS)

**DOI:** 10.2188/jea.JE20140028

**Published:** 2014-11-05

**Authors:** Takehiko Tsujimoto, Toshimi Sairenchi, Hiroyasu Iso, Fujiko Irie, Kazumasa Yamagishi, Hiroshi Watanabe, Kiyoji Tanaka, Takashi Muto, Hitoshi Ota

**Affiliations:** 1 Faculty of Health and Sport Sciences, University of Tsukuba, Ibaraki, Japan; 1 筑波大学体育系; 2 Ibaraki Health Plaza, Ibaraki Health Service Association, Ibaraki, Japan; 2 茨城県立健康プラザ; 3 Department of Public Health, Dokkyo Medical University School of Medicine, Tochigi, Japan; 3 獨協医科大学医学部公衆衛生学講座; 4 Department of Social and Environmental Medicine, Graduate School of Medicine, Osaka University, Osaka, Japan; 4 大阪大学大学院医学系研究科社会医学専攻公衆衛生学; 5 Department of Health and Welfare, Ibaraki Prefectural Office, Ibaraki, Japan; 5 茨城県保健福祉部保健予防課; 6 Department of Public Health Medicine, Faculty of Medicine, University of Tsukuba, Ibaraki, Japan; 6 筑波大学医学医療系社会健康医学; 7 Ibaraki Health Service Association, Ibaraki, Japan; 7 茨城県総合健診協会

**Keywords:** chronic kidney disease, body mass index, obesity, dose-response relationship, epidemiology

## Abstract

**Purpose:**

To examine the relationship between body mass index (BMI) and the risk of stage ≥3 chronic kidney disease (CKD) in a general Japanese population.

**Methods:**

A total of 105 611 participants aged 40–79 years who completed health checkups in Ibaraki Prefecture, Japan, and were free of CKD in 1993 were followed-up through 2006. Stage ≥3 CKD was defined by an estimated glomerular filtration rate <60 mL/min/1.73 m^2^ reported during at least 2 successive annual surveys or as treatment for kidney disease. Hazard ratios (HRs) for the development of stage ≥3 CKD relative to the BMI categories were calculated using the Cox proportional hazards regression model, which was adjusted for possible confounders and mediators.

**Results:**

During a mean follow-up of 5 years, 19 384 participants (18.4%) developed stage ≥3 CKD. Compared to a BMI of 21.0–22.9 kg/m^2^, elevated multivariable-adjusted HRs were observed among men with a BMI ≥23.0 kg/m^2^ and women with a BMI ≥27.0 kg/m^2^. Significant dose-response relationships between BMI and the incidence of stage ≥3 CKD were observed in both sexes (*P* for trend <0.001).

**Conclusions:**

Obesity was associated with the risk of developing stage ≥3 CKD among men and women.

## Abstract (ja)

**【目的】:**

日本人におけるbody mass index（BMI）とステージ3以上の慢性腎臓病発症リスクとの関連を検討すること。

**【方法】:**

対象者は、茨城県内における1993年の基本健康診査の受診者で慢性腎臓病の既往歴のない40～74歳の男女105611人であった。対象者の慢性腎臓病発症を2006年まで追跡した。ステージ3以上の慢性腎臓病発症の定義は推定糸球体濾過量が2回以上の健診で60 ml/min/1.73 m^2^未満となった時点、もしくは腎臓病治療開始時点とした。 BMI区分（18.5未満、18.5～20.9、21.0～22.9、23.0～24.9、25.0～26.9、27.0～29.9、30.0以上）の慢性腎臓病発症に対する多変量調整ハザード比はCoxの比例ハザードモデルを用いて算出し、可能性のある交絡要因で調整した。

**【結果】:**

平均5年の追跡期間中に19384人（男性6283人、女性14015人）のステージ3以上の慢性腎臓病発症が確認された。ステージ3以上の慢性腎臓病発症に対する多変量調整ハザード比は、BMIが21.0～22.9を基準とした場合、男性でBMIが23.0以上、女性でBMIが27.0以上で有意に高値を示した。また、男女ともに、BMIとステージ3以上の慢性腎臓病発症率との間に量・反応関係が認められた。

**【まとめ】:**

肥満は、男女ともにステージ3以上の慢性腎臓病の発症リスクと関連する。

## INTRODUCTION

Chronic kidney disease (CKD) is a major public health problem. In Japan, CKD affects 13.3 million adults.^1^ With the increasing incidence of hypertension and type 2 diabetes and the aging of the Japanese population, the number of individuals with CKD will likely continue to increase. CKD is recognized as an independent risk factor for myocardial infarction and cardiovascular mortality and can result in significant morbidity, mortality, and increased medical costs.^2^

Obesity is also a major public health issue, and its prevalence has been increasing worldwide. Obesity is associated with the development of many cardiovascular disease (CVD) risk factors, including type 2 diabetes mellitus,^3^^,^^4^ hypertension,^5^^,^^6^ dyslipidemia,^7^ and CKD.^8^ Prospective cohort studies have revealed the longitudinal relation between body mass index (BMI) and the risk of moderate CKD. A greater baseline BMI was associated with an increased risk of stage ≥3 CKD in the Physician’s Health Study,^9^ the Hypertension Detection and Follow-Up Program,^10^ and the Framingham Heart Study.^11^ Because treatment of long-term CKD is costly, the best approach is to reduce the incidence of stage ≥3 CKD or prevent it entirely. Examining the modifiable risk factors for stage ≥3 CKD, such as obesity, is important because of the public health implications.

A relationship between obesity and the risk of stage ≥3 CKD in Japanese participants has been reported.^12^ However, not enough information was presented to examine the dose-response relationship between obesity and the risk of CKD (ie obesity was only considered as dichotomous data); consequently, the dose-response relationship in Japanese individuals remains unclear. An examination of the CKD risk using more-detailed BMI categories in a large cohort is warranted. Additionally, no studies have considered the age-specific relationship between BMI and the development of stage ≥3 CKD. Further research on this issue may help officials implement more effective public health and clinical efforts aimed at the primary prevention of CKD. The purpose of our study was to examine the dose-response relationship between BMI and the development of stage ≥3 CKD in a general Japanese population.

## METHODS AND PROCEDURES

### Study population

The study population consisted of 194 333 individuals (63 865 men and 130 468 women) aged 40–79 years who were living in Ibaraki Prefecture, Japan. These individuals had participated in community-based annual health checkups in 1993 (as part of the Ibaraki Prefectural Health Study), which were conducted by the local governments in accordance with the Law of Health and Medical Services for the Elderly. The Ibaraki prefectural government collected data from the local governments, and personal information was removed to ensure anonymity. We excluded 18 939 patients (2367 men and 16 572 women) because of incomplete data, 10 075 individuals (4101 men and 5974 women) because of a history of CVD, and 10 491 individuals (3615 men and 6876 women) because of the presence of stage ≥3 CKD and/or ongoing treatment for CKD. We further excluded 48 864 individuals (17 999 men and 30 865 women) who failed to participate in the 1994 survey, thereby ensuring that all of the participants were followed for at least one year.

Ultimately, the study included 105 611 participants (35 738 men and 69 873 women). These participants were followed by annual examinations until a diagnosis of stage ≥3 CKD, withdrawal from the repeated examinations, or the end of 2006, whichever occurred first. The Ibaraki Epidemiology Study Union Ethics Review Committee approved the protocol for this cohort study.

### Measurements

Kidney function was assessed using the estimated glomerular filtration rate (eGFR). The eGFR was calculated using the new Japanese abbreviated prediction equation,^13^ modified from the Modification of Diet in Renal Disease (MDRD) Study,^14^ as recommended by the Japanese Society of Nephrology:

$$\begin{array}{ll} & \text{eGFR}(\text{mL}/[\text{min}\cdot 1.73m^{2}])\\ & \;={\left[194\times (\text{serum creatinine}[\text{mg}/\text{dL}])\right]}^{-1.094}\\ & \;\times {\left(\text{age}\right)}^{-0.287}\times 0.739(\text{for women only}) \end{array}$$

According to Levey et al, stage ≥3 CKD is defined as the presence of kidney damage or an eGFR <60 mL/min/1.73 m^2^ reported at least twice in successive annual surveys.^15^

Serum creatinine level was measured using the Jaffe method with an automated analyzer (Hitachi 7350; Hitachi, Tokyo, Japan, or RX-30; Nihon Denshi, Tokyo, Japan) in 1993–2003; in 2004–2006, it was measured using the enzyme method with an automated analyzer (Hitachi 7770; Hitachi). The coefficient of validation for creatinine value was 0.61%. Serum creatinine measurements from 1993–2003 were converted to the value obtained in the enzyme method using the following equation:

$$\begin{array}{ll} & \text{serum creatinine by enzyme method (mg/dL)}\\ & \;=0.9915\times \text{serum creatinine by the Jaffe method (mg/dL)}\\ & \;-0.211 \end{array}$$

The serum creatinine values measured using the enzyme method and the serum creatinine values measured using the Jaffe method were then converted to the enzyme method from the same subjects at the same point in time, and the comparability between them was found to be excellent (*r* = 0.99, *P* < 0.001). Proteinuria was defined as a urinary protein excretion of 1+ or more by dipstick test (Ames Hemacombisticks; Bayer-Sankyo Ltd., Tokyo, Japan).

The patients’ height in sock feet and weight in light clothing were measured at baseline. BMI was calculated as the weight in kilograms divided by the height in meters squared (kg/m^2^).

We measured the following cardiovascular risk factors: serum total cholesterol, serum high-density-lipoprotein (HDL) cholesterol, serum triglyceride, plasma glucose, blood pressure, use of medications, cigarette smoking, and typical alcohol intake. Blood samples were drawn into two polyethylene terephthalate tubes from seated participants; one tube contained an accelerator, while the other contained sodium fluoride and ethylenediaminetetraacetic acid. Overnight fasting (≥8 h) was not mandatory. The serum total cholesterol and serum triglyceride levels were measured using the enzyme method with the RX-30 device in 1993–1995, the H7350 device in 1996–2003, and the H7700 device in 2004–2006. The HDL cholesterol levels were measured using the phosphotungstic acid magnesium method with an MTP-32 device (Corona Electric, Ibaraki, Japan) in 1993–1995, the selective inhibition method with the H7350 device in 1996–2003, and the H7700 device in 2004–2006. Dyslipidemia was defined as triglycerides ≥1.7 mmol/L, HDL cholesterol <1.036 mmol/L, or as the patient being prescribed medication for dyslipidemia treatment.

The blood glucose level was measured using the glucose oxidase electrode method with a GA1140 device (Kyoto Daiichi Kagaku, Kyoto, Japan) in 1993–1996, the enzyme method with a H7170 device (Hitachi) in 1997–2003, and the H7700 device in 1994–2006. The participants were considered diabetic if they had a plasma glucose of ≥6.1 mmol/L in a fasted state or ≥7.8 mmol/L in a non-fasted state, or if they were being treated for diabetes mellitus. The laboratory participated in external standardization and successfully met the criteria for precision accuracy for the measurement of blood samples, as established by the Japan Medical Association, the Japanese Association of Medical Technologists, and the Japan Society of Health Evaluation and Promotion.

Blood pressure was measured on the right arm of seated participants who had rested for more than 5 min; trained observers obtained these measurements using a standard mercury sphygmomanometer in 1993–2004 and an automated sphygmomanometer in 2005–2006. When the systolic blood pressure was >150 mm Hg or the diastolic blood pressure was >90 mm Hg, a second measurement was obtained after the subject took several deep breaths. The lower values, which were almost always observed during the second measurement, were used for the analyses. Hypertension was defined as systolic blood pressure ≥140 mm Hg, diastolic blood pressure ≥90 mm Hg, or use of antihypertensive medication. CVD risk factors were defined as hypertension, dyslipidemia, and diabetes.

Lastly, we conducted an interview to ascertain the number of cigarettes smoked per day, the typical weekly alcohol intake (converted to grams of ethanol per day), and the history of CVD and CKD.

### Statistical analysis

The participants were classified into the following categories with regard to their BMI (kg/m^2^): <18.5; 18.5–20.9; 21.0–22.9; 23.0–24.9; 25.0–26.9; 27.0–29.9; or ≥30.0. To compare the participants’ physical characteristics according to the BMI categories, one-way analysis of variance was used for continuous variables, and a χ^2^-test was used for categorical variables. The Cox proportional hazards regression model was used to calculate hazard ratios (HRs) and the 95% confidence intervals (CIs) of risk of development of stage ≥3 CKD relative to the BMI categories in comparison to the reference group, 21.0–22.9 kg/m^2^. A BMI of 22 kg/m^2^ is commonly set as the optimal body size in Japan.^16^ The analyses were stratified by sex and age groups (40–59 and 60–79 years old).

We used two multivariate-adjusted models. In model one, covariates included age and the potential confounders of cigarette smoking (never, former, current [1–19 cigarettes/day or ≥20 cigarettes/day]) and typical alcohol intake (never, sometimes, everyday [<56 g/day or ≥56 g/day]). In model two, potential mediators were added to model one. Potential mediators included systolic blood pressure, the use of antihypertensive medication (yes or no), triglyceride level (log-transformed), serum total cholesterol, serum HDL cholesterol, the use of lipid medication (yes or no), blood glucose status (normal [<6.1 mmol/L in a fasted state or <7.8 mmol/L in a non-fasted state], borderline [6.1–7.0 mmol/L in a fasted state or 7.8–11.1 mmol/L in a non-fasted state], hyperglycemic [>7.0 mmol/L in a fasted state or >11.1 mmol/L in a non-fasted state]), the use of diabetes medication (yes or no), and proteinuria (yes or no). A *P* value <0.05 was regarded as statistically significant. The SAS System for Windows, release 9.3 (SAS Institute Inc., Cary, NC, USA), was used for all analyses.

## RESULTS

Sex-stratified baseline characteristics of the cardiovascular risk factors according to our BMI categories are provided in Table 1. All of the factors, except diabetic medication use in men and lipid medication use in men and women, were associated with BMI in both sexes. A higher BMI was linked with a higher eGFR and a higher prevalence of proteinuria in both sexes.

**Table 1.** Baseline characteristics of participants by BMI categories

Gender and baseline variables	Body mass index, kg/m^2^							*P* for difference

	<18.5	18.5–20.9	21.0–22.9	23.0–24.9	25.0–26.9	27.0–29.9	≥30.0	
Men (*n* = 35 738)								
Number of participants	1570	6717	9044	9097	5928	2899	483	
Age, years	65.0 (8.8)	62.5 (9.5)	60.8 (9.7)	59.8 (9.7)	59.0 (9.6)	58.9 (9.4)	57.4 (9.4)	<0.001
eGFR, mL/(min·1.73 m^2^)	89.9 (18.6)	90.2 (18.4)	88.6 (17.4)	87.1 (17.3)	86.4 (16.8)	85.3 (16.5)	84.4 (16.6)	<0.001
Proteinuria, %	2.2	1.7	1.4	1.8	2.2	3.7	6.4	<0.001
Total cholesterol, mmol/L	4.67 (0.81)	4.75 (0.82)	4.94 (0.85)	5.07 (0.86)	5.17 (0.86)	5.21 (0.86)	5.26 (0.86)	<0.001
HDL cholesterol, mmol/L	1.63 (0.43)	1.52 (0.40)	1.41 (0.38)	1.30 (0.34)	1.24 (0.31)	1.18 (0.29)	1.14 (0.28)	<0.001
Triacylglycerol, mmol/L	1.06 (0.59)	1.21 (0.71)	1.50 (0.91)	1.78 (1.05)	2.05 (1.22)	2.23 (1.31)	2.32 (1.31)	<0.001
Blood glucose, mmol/L	6.41 (2.15)	6.37 (2.09)	6.35 (1.98)	6.39 (2.03)	6.45 (2.01)	6.60 (2.26)	6.70 (2.23)	<0.001
Systolic blood pressure, mm Hg	131.4 (18.2)	133.5 (17.7)	135.1 (16.9)	136.9 (16.6)	138.2 (16.2)	140.8 (16.7)	142.6 (16.2)	<0.001
Diastolic blood pressure, mm Hg	76.9 (10.6)	78.1 (10.4)	79.7 (10.3)	81.3 (10.3)	82.9 (10.3)	84.6 (10.6)	86.9 (10.9)	<0.001
Lipid medication use, %	0.4	0.7	1.2	1.6	1.5	2.0	2.3	0.289
Diabetic medication use, %	3.2	2.6	2.7	3.6	3.7	4.0	3.1	0.361
Antihypertensive medication use, %	12.5	14.5	16.6	19.7	22.4	26.5	32.1	<0.001
Smoking status, %								<0.001
Never	18.1	18.7	22.3	24.1	24.4	25.1	28.6	
Former	22.2	23.5	27.3	30.3	32.0	33.3	28.8	
Current								
<20 cigarettes/day	26.4	21.0	16.2	13.8	12.0	10.7	9.7	
≥20 cigarettes/day	33.3	36.8	34.2	31.9	31.6	31.0	32.9	
Alcohol intake, %								<0.001
Never	44.8	35.6	31.9	31.1	31.0	33.4	37.7	
Sometimes	10.4	11.1	11.9	13.8	14.6	15.7	14.9	
Everyday								
<56 g/day	41.1	47.5	49.5	49.1	47.3	42.9	38.9	
≥56 g/day	3.6	5.8	6.7	6.1	7.1	8.1	8.5	
Women (*n* = 69 873)								
Number of participants	2846	12 052	17 146	17 122	11 559	7229	1919	
Age, years	60.4 (10.3)	57.5 (9.8)	57.8 (9.3)	58.5 (8.8)	59.4 (8.6)	59.8 (8.4)	59.0 (8.5)	<0.001
eGFR, mL/(min·1.73 m^2^)	94.5 (22.0)	96.1 (22.3)	94.2 (21.1)	93.4 (24.9)	91.8 (20.7)	91.4 (20.4)	91.3 (21.0)	<0.001
Proteinuria, %	0.9	0.7	0.8	0.9	1.2	1.7	3.3	<0.001
Total cholesterol, mmol/L	5.19 (0.88)	5.27 (0.87)	5.39 (0.89)	5.48 (0.89)	5.55 (0.88)	5.60 (0.91)	5.61 (0.92)	<0.001
HDL cholesterol, mmol/L	1.72 (0.40)	1.61 (0.38)	1.51 (0.36)	1.43 (0.34)	1.38 (0.33)	1.35 (0.31)	1.33 (0.31)	<0.001
Triacylglycerol, mmol/L	1.07 (0.50)	1.23 (0.65)	1.42 (0.79)	1.61 (0.90)	1.77 (0.96)	1.88 (1.04)	1.94 (0.99)	<0.001
Blood glucose, mmol/L	5.90 (1.61)	5.79 (1.40)	5.83 (1.40)	5.96 (1.50)	6.04 (1.52)	6.16 (1.71)	6.35 (2.03)	<0.001
Systolic blood pressure, mm Hg	126.5 (17.9)	127.2 (17.3)	130.1 (17.0)	132.7 (16.9)	135.4 (16.6)	138.6 (16.8)	141.7 (16.9)	<0.001
Diastolic blood pressure, mm Hg	73.5 (10.4)	74.7 (10.3)	76.4 (10.1)	78.3 (10.0)	79.9 (10.0)	82.0 (10.0)	84.1 (10.6)	<0.001
Lipid medication use, %	1.9	2.2	3.2	3.8	4.2	4.6	4.6	0.988
Diabetic medication use, %	1.3	1.5	1.5	1.9	2.3	2.7	3.2	<0.001
Antihypertensive medication use, %	8.7	10.4	13.8	19.0	23.9	30.6	38.0	<0.001
Smoking status, %								<0.001
Never	92.2	95.1	95.5	95.9	95.7	95.2	93.3	
Former	0.5	0.4	0.6	0.5	0.6	0.7	0.8	
Current								
<20 cigarettes/day	4.9	3.2	2.7	2.4	2.5	2.7	3.9	
≥20 cigarettes/day	2.3	1.3	1.1	1.3	1.3	1.5	2.1	
Alcohol intake, %								<0.001
Never	91.3	90.0	90.0	90.7	90.8	91.7	91.5	
Sometimes	4.8	5.8	6.3	5.7	5.9	5.1	4.8	
Everyday								
<56 g/day	3.9	4.1	3.6	3.5	3.2	3.1	3.4	
≥56 g/day	—	0.1	0.1	0.1	0.1	0.1	0.3	

BMI, body mass index; eGFR, estimated glomerular filtration rate; HDL, high-density lipoprotein; SD, standard deviation.

Showing mean (SD) for continuous variables: age, fasting and non-fasting blood glucose, systolic and diastolic blood pressure, total cholesterol, HDL cholesterol, and triglycerides.

SI conversion factors: to convert blood glucose values to mmol/L, multiply by 0.055 51; to convert cholesterols values to mmol/L, multiply by 0.025 86; to convert triglycerides values to mmol/L, multiply by 0.011 29.

Of the 105 611 participants (35 738 men and 69 873 women), 19 384 (18.4%) developed stage ≥3 CKD (5978 men and 13 406 women) over a mean follow-up of 5 years (4.9 years for men and 5.1 years for women). Table 2 and Figure show the sex-stratified HRs for the incidence of stage ≥3 CKD according to BMI category. In both sexes, compared to a BMI of 21.0–22.9 kg/m^2^, the age- and potential confounder-adjusted HRs were higher for the higher BMI categories (model 1; *P* for trend <0.001; Table 2). Further, these results were similar even when adjusted for potential mediators (model 2; Figure). The HRs of BMI ≥30.0 kg/m^2^ were markedly higher in men and women (HR 1.60, 95% CI 1.24–2.06 and HR 1.41, 95% CI 1.25–1.60, respectively).

**Figure.**
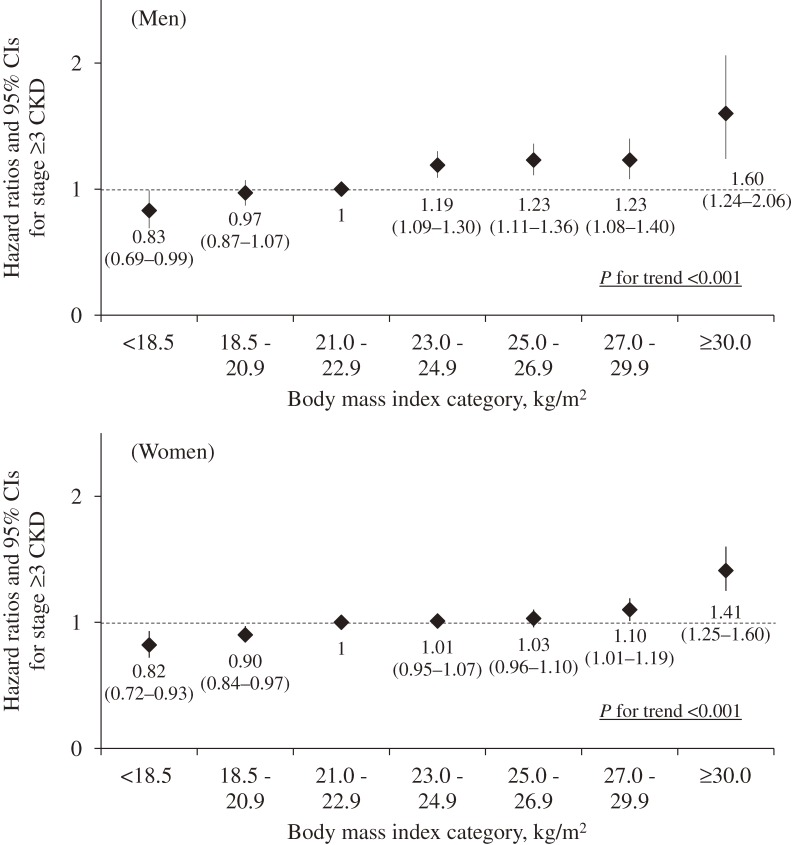
The multivariable-adjusted hazard ratios (HRs) and 95% confidence intervals (CIs) for the development of stage ≥3 chronic kidney disease (CKD) in men and women.

**Table 2.** Sex-specific HRs and 95% CI for stage ≥3 CKD by BMI categories

Sex and body mass index category (kg/m^2^)	Number of participants	Number of person-years	Incidence rates per 1000 person-years	Age-adjusted HR	95% CI	Multivariate- adjusted HR^a^ (model 1)	95% CI	*P* for trend
Men								
<18.5	1570	7061	20.1	**0.76**	**0.63, 0.90**	**0.73**	**0.69, 0.61**	
18.5–20.9	6717	33 677	20.1	**0.90**	**0.82, 0.99**	**0.89**	**0.87, 0.81**	
21.0–22.9	9044	47 022	19.9	1	(ref.)	1	(ref.)	
23.0–24.9	9097	46 973	23.1	**1.27**	**1.16, 1.38**	**1.27**	**1.09, 1.17**	<0.001
25.0–26.9	5928	30 170	22.9	**1.38**	**1.25, 1.52**	**1.39**	**1.11, 1.26**	
27.0–29.9	2899	14 091	23.6	**1.48**	**1.31, 1.68**	**1.48**	**1.08, 1.30**	
≥30.0	483	2252	28.4	**2.01**	**1.56, 2.59**	**1.98**	**1.24, 1.54**	
Women								
<18.5	2846	14 223	19.8	**0.75**	**0.66, 0.85**	**0.74**	**0.72, 0.66**	
18.5–20.9	12 052	65 680	17.8	**0.86**	**0.80, 0.93**	**0.86**	**0.84, 0.80**	
21.0–22.9	17 146	94 954	20.1	1	(ref.)	1	(ref.)	
23.0–24.9	17 122	92 420	22.0	1.05	0.99, 1.12	1.05	0.95, 0.99	<0.001
25.0–26.9	11 559	61 186	24.7	**1.11**	**1.04, 1.19**	1.11	0.96, 1.04	
27.0–29.9	7229	36 348	27.9	**1.23**	**1.14, 1.33**	**1.23**	**1.01, 1.14**	
≥30.0	1919	8760	34.5	**1.66**	**1.47, 1.87**	**1.64**	**1.25, 1.45**	

BMI, body mass index; CI, confidence interval; CKD, chronic kidney disease; HR, hazard ratio.

^a^Adjusted for age (years), smoking status (never, ex-, current <20 cigarettes/day, or ≥20 cigarettes/day), and alcohol intake (never, sometimes, <56 g/day, or ≥56 g/day).

Table 3 shows the sex-stratified HRs for stage ≥3 CKD by BMI categories among diabetes-free and CVD risk factor-free patients at baseline. In analyses limited to those free of either diabetes or of any CVD risk factors, the HRs were higher for the higher BMI categories (*P* for trend <0.001).

**Table 3.** Sex-specific HRs and 95% CIs for stage ≥3 CKD by BMI categories among diabetes-free and CVD risk factor-free at baseline

Sex and body mass index category (kg/m^2^)	Number of subjects	Number of person-years	Incidence rates per 1000 person-years	Age-adjusted HR	95% CI	Multivariable HR^c^	95% CI	*P* for trend
**Diabetes**^a^**-free**								
**Men**								
<18.5	1207	5426	19.5	**0.73**	**0.60, 0.90**	**0.80**	**0.65, 0.99**	
18.5–20.9	5352	26 978	19.4	**0.89**	**0.79, 0.99**	0.94	0.84, 1.05	
21.0–22.9	7247	38 202	19.9	1	(ref.)	1	(ref.)	
23.0–24.9	7255	37 952	23.1	**1.27**	**1.15, 1.40**	**1.21**	**1.09, 1.33**	<0.001
25.0–26.9	4601	23 495	23.0	**1.38**	**1.24, 1.55**	**1.26**	**1.12, 1.41**	
27.0–29.9	2227	11 013	22.2	**1.44**	**1.25, 1.67**	**1.24**	**1.07, 1.44**	
>30.0	359	1715	29.2	**2.02**	**1.52, 2.69**	**1.64**	**1.23, 2.19**	
**Women**								
<18.5	2492	12 512	19.4	**0.75**	**0.65, 0.86**	**0.80**	**0.70, 0.92**	
18.5–20.9	10 818	58 756	17.3	**0.86**	**0.79, 0.93**	**0.89**	**0.82, 0.96**	
21.0–22.9	15 336	85 182	19.8	1	(ref.)	1	(ref.)	
23.0–24.9	15 086	81 737	21.4	1.03	0.97, 1.11	1.00	0.93, 1.06	<0.001
25.0–26.9	10 024	53 502	24.4	**1.11**	**1.03, 1.19**	1.04	0.96, 1.11	
27.0–29.9	6182	31 475	27.2	**1.21**	**1.12, 1.32**	**1.09**	**1.00, 1.18**	
>30.0	1574	7272	33.7	**1.65**	**1.44, 1.89**	**1.41**	**1.23, 1.62**	
**CVD risk factor**^b^**-free**								
**Men**								
<18.5	501	2364	11.8	**0.63**	**0.42, 0.95**	**0.64**	**0.42, 0.96**	
18.5–20.9	1815	9989	12.0	0.83	0.65, 1.06	0.84	0.65, 1.08	
21.0–22.9	1945	10 918	12.4	1	(ref.)	1	(ref.)	
23.0–24.9	1528	8402	13.9	**1.29**	**1.01, 1.65**	**1.30**	**1.01, 1.67**	<0.001
25.0–26.9	757	4007	12.5	1.36	0.98, 1.88	1.30	0.93, 1.81	
27.0–29.9	232	1121	16.1	**1.85**	**1.13, 3.03**	**1.78**	**1.08, 2.93**	
>30.0	29	109	9.2	2.20	0.31, 15.75	1.94	0.27, 13.92	
**Women**								
<18.5	1304	6802	15.1	0.90	0.72, 1.12	0.92	0.74, 1.15	
18.5–20.9	5285	29 349	12.0	0.99	0.86, 1.14	1.01	0.87, 1.16	
21.0–22.9	6243	36 399	11.8	1	(ref.)	1	(ref.)	
23.0–24.9	4920	27 907	14.0	**1.17**	**1.02, 1.34**	**1.16**	**1.01, 1.33**	<0.001
25.0–26.9	2509	14 196	16.3	**1.27**	**1.08, 1.49**	**1.21**	**1.03, 1.42**	
27.0–29.9	1126	5989	19.2	**1.57**	**1.27, 1.92**	**1.47**	**1.2, 1.81**	
>30.0	222	1111	26.1	**2.15**	**1.47, 3.13**	**1.98**	**1.35, 2.89**	

BMI, body mass index; CI, confidence interval; CKD, chronic kidney disease; CVD, cardiovascular disease; HR, hazard ratio.

^a^Diabetes is defined as plasma glucose ≥6.1 mmol/L in a fasted state or ≥7.8 mmol/L in a non-fasted state, or being treated for diabetes mellitus.

^b^Cardiovascular disease risk factors are hypertension, dyslipidemia, and diabetes.

^c^Adjusted for age (years), systolic blood pressure (mm Hg), total cholesterol level (mmol/liter), high-density lipoprotein cholesterol level (mmol/liter), log-transformed triglyceride level (mmol/liter), proteinuria (yes or no), smoking status (never, ex-, current <20 cigarettes/day, or ≥20 cigarettes/day), and alcohol intake (never, sometimes, <56 g/day, or ≥56 g/day).

Table 4 shows the sex- and age-stratified HRs for the incidence of stage ≥3 CKD by BMI category compared with a BMI of 21.0–22.9 kg/m^2^. In men aged 40–59 years, the multivariable HRs of BMI ≥30.0 kg/m^2^ were significantly higher. In men aged 60–79 years, the multivariable HRs of BMI ≥23.0 kg/m^2^ were significantly higher. In women aged 40–59 years, the multivariable HRs of the overall BMI categories were not significantly associated (*P* for trend = 0.291). In women aged 60–79 years, the multivariable HRs of BMI ≥27.0 kg/m^2^ were significantly higher. In both sexes and age classes, except women aged 40–59 years, a significant dose-response relationship between BMI and the incidence of stage ≥3 CKD was observed.

**Table 4.** Age and sex-specific HRs and 95% CIs for stage ≥3 CKD by BMI categories

Sex, age group, and BMI categories (kg/m^2^)	Number of participants	Number of person-years	Incidence rates per 1000 person-years	Age-adjusted HR	95% CI	Multivariable HR^a^	95% CI	*P* for trend
Men								
Age 40–59 years								
<18.5	298	1373	2.9	0.44	0.16, 1.18	0.47	0.17, 1.28	
18.5–20.9	1948	10 900	4.3	**0.63**	**0.45, 0.88**	**0.71**	**0.50, 0.99**	
21.0–22.9	3310	18 194	6.7	1	(ref.)	1	(ref.)	
23.0–24.9	3762	20 086	9.4	**1.41**	**1.12, 1.77**	1.22	0.97, 1.54	0.001
25.0–26.9	2676	14 323	8.9	**1.34**	**1.05, 1.72**	1.10	0.85, 1.42	
27.0–29.9	1384	7130	10.9	**1.57**	**1.18, 2.09**	1.21	0.90, 1.63	
≥30.0	264	1241	16.9	**2.59**	**1.63, 4.11**	**1.83**	**1.14, 2.95**	
Age 60–79 years								
<18.5	1272	5688	24.3	**0.77**	**0.64, 0.92**	0.84	0.70, 1.01	
18.5–20.9	4769	22 777	27.7	0.93	0.84, 1.03	0.99	0.89, 1.10	
21.0–22.9	5734	28 828	28.3	1	(ref.)	1	(ref.)	
23.0–24.9	5335	26 887	33.4	**1.25**	**1.13, 1.37**	**1.18**	**1.07, 1.30**	<0.001
25.0–26.9	3252	15 847	35.5	**1.39**	**1.25, 1.55**	**1.26**	**1.13, 1.41**	
27.0–29.9	1515	6961	36.6	**1.46**	**1.27, 1.68**	**1.22**	**1.06, 1.41**	
≥30.0	219	1011	42.5	**1.83**	**1.34, 2.48**	**1.47**	**1.08, 2.01**	
Women								
Age 40–59 years								
<18.5	1209	6864	6.8	0.91	0.68, 1.23	0.98	0.72, 1.33	
18.5–20.9	6611	38 770	6.7	0.91	0.78, 1.07	0.96	0.82, 1.12	
21.0–22.9	9324	56 401	7.7	1	(ref.)	1	(ref.)	
23.0–24.9	8808	51 514	8.4	1.04	0.91, 1.19	1.00	0.88, 1.15	0.291
25.0–26.9	5436	31 685	9.2	1.10	0.95, 1.28	1.02	0.87, 1.18	
27.0–29.9	3219	17 926	9.5	1.17	0.98, 1.39	1.02	0.85, 1.22	
≥30.0	928	4778	11.3	**1.46**	**1.10, 1.94**	1.19	0.90, 1.59	
Age 60–79 years								
<18.5	1637	7359	31.9	**0.75**	**0.65, 0.86**	**0.81**	**0.70, 0.93**	
18.5–20.9	5441	26 910	33.9	**0.86**	**0.79, 0.93**	**0.89**	**0.82, 0.97**	
21.0–22.9	7822	38 553	38.3	1	(ref.)	1	(ref.)	
23.0–24.9	8314	40 906	39.1	1.04	0.97, 1.12	1.00	0.93, 1.07	<0.001
25.0–26.9	6123	29 501	41.4	**1.09**	**1.01, 1.18**	1.02	0.95, 1.10	
27.0–29.9	4010	18 422	45.8	**1.22**	**1.12, 1.33**	**1.10**	**1.00, 1.19**	
≥30.0	991	3982	62.3	**1.67**	**1.46, 1.90**	**1.44**	**1.26, 1.65**	

BMI, body mass index; CI, confidence interval; CKD, chronic kidney disease; HR, hazard ratio.

^a^Adjusted for age (years), smoking status (never, ex-, current <20 cigarettes/day, or ≥20 cigarettes/day), alcohol intake (never, sometimes, <56 g/day, or ≥56 g/day), fasting status (yes or no), systolic blood pressure (mm Hg), antihypertensive medication use (yes or no), total cholesterol level (mmol/liter), high-density lipoprotein cholesterol level (mmol/liter), log-transformed triglyceride level (mmol/liter), lipid medication use (yes or no), blood glucose status (normal: <6.1 mmol/l during fasting or <7.8 mmol/l during nonfasting; border: 6.1–7.0 mmol/l during fasting or 7.8–11.1 mmol/l during nonfasting; hyperglycemic: 7.0 mmol/l during fasting or 11.1 mmol/l during nonfasting), diabetes medication use (yes or no), and proteinuria (yes or no).

## DISCUSSION

To the best of our knowledge, this is the first cohort study to demonstrate a dose-response relationship between obesity and the risk of stage ≥3 CKD in a Japanese population. The dose-response relationship was found in men aged 40–59 and 60–79 years and in women aged 60–79 years. In addition, this relationship was independent of diabetes and other CVD risk factors (ie hypertension and dyslipidemia). We also observed that the risk of stage ≥3 CKD was markedly higher in obese men and women with a BMI ≥30.0 kg/m^2^ than in men and women with a BMI of 21.0–22.9 kg/m^2^, except in women aged 40–59 years.

The significant relationship observed between BMI and the incidence of stage ≥3 CKD in our study was consistent with that observed in previous studies in Caucasian and Asian populations.^11^^,^^17^^,^^18^ The Framingham Offspring Study, which included 2585 participants (mean age, 43 years) who were followed from 1978–2001 (mean follow-up, 18.5 years), showed a strong dose-response relationship between baseline BMI and risk of CKD (defined as eGFR using the MDRD Study equation: ≤64.25 mL/[min·1.73 m^2^] in men and ≤59.25 mL/[min·1.73 m^2^] in women).^11^ The multivariable odds ratio of CKD was 1.23 (95% CI, 1.08–1.41) per one standard deviation of approximately 4 kg.^11^ A Japanese community-based study, which followed 100 753 individuals (mean age, 49 years) for 17 years, revealed that a higher BMI at baseline was associated with an increased risk of end-stage renal disease in men but not women.^18^ The multivariable-adjusted odds ratios of end-stage renal disease were 1.27 (95% CI, 1.21–1.45) in men and 0.95 (95% CI, 0.83–1.09) in women for each 2 kg/m^2^ increment of BMI.^18^ Although these authors did not examine the association between BMI and the risk of CKD among older adults, their results in middle-aged adults are consistent with our findings.

The association between obesity and stage ≥3 CKD may be mediated through multiple biological mechanisms, including hormonal factors, inflammation, oxidative stress, and endothelial dysfunction.^19^^,^^20^ In obese individuals, the rennin-angiotensin-aldosterone system is commonly activated,^21^ and it is a well-coordinated hormonal system that regulates adrenal, cardiovascular, and kidney function by controlling the fluid and electrolyte balance. Activation of this system leads to the development of hypertension via the production of angiotensin 2, which causes further damage to the kidneys.^22^ Estrogen, a sex hormone that is secreted more in premenopausal women compared with men and postmenopausal women, decreases the expression of angiotensin type 1 receptors in the vasculature and kidneys^23^ and reduces the expression and activity of angiotensin-converting enzymes.^24^^,^^25^ These biological mechanisms may be underlying factors for the significant relationship between obesity and the development of stage ≥3 CKD among middle-aged women in our study.

The strength of our study is that stage ≥3 CKD was defined as an eGFR level <60 mL/min/1.73 m^2^ reported at more than two successive annual surveys. Further, all of the blood samples were measured by the same laboratory, which was verified using a validated quality control system.^26^ However, there are several limitations. First, we only examined generalized obesity and not abdominal obesity, because the measurements of central obesity were not available during the baseline examination. Second, potential residual confounders may not have been assessed, such as fat distribution, dietary lifestyle (ie protein and salt intake), and physical activity. Third, detailed information on use of medications such as statins and omega 3-fatty acids was not collected because of the nature of the community-based health checkup.

Obesity was associated with the risk of developing stage ≥3 CKD among men and older women. Compared with participants who had a normal BMI (21.0–22.9 kg/m^2^), those with a BMI ≥30.0 kg/m^2^ had a markedly high risk of developing stage ≥3 CKD. Weight management may be important for preventing CKD in obese men and women.

## ONLINE ONLY MATERIAL

Abstract in Japanese.

## References

[1] Imai E, Horio M, Watanabe T, Iseki K, Yamagata K, Hara S, . Prevalence of chronic kidney disease in the Japanese general population. Clin Exp Nephrol. 2009;13:621–30. 10.1007/s10157-009-0199-x 19513802

[2] Weiner DE, Tabatabai S, Tighiouart H, Elsayed E, Bansal N, Griffith J, . Cardiovascular outcomes and all-cause mortality: exploring the interaction between CKD and cardiovascular disease. Am J Kidney Dis. 2006;48:392–401. 10.1053/j.ajkd.2006.05.021 16931212

[3] Weinstein AR, Sesso HD, Lee IM, Cook NR, Manson JE, Buring JE, . Relationship of physical activity vs body mass index with type 2 diabetes in women. JAMA. 2004;292:1188–94. 10.1001/jama.292.10.1188 15353531

[4] Sasai H, Sairenchi T, Iso H, Irie F, Otaka E, Tanaka K, . Relationship between obesity and incident diabetes in middle-aged and older Japanese adults: the Ibaraki Prefectural Health Study. Mayo Clin Proc. 2010;85:36–40. 10.4065/mcp.2009.0230 20042559 PMC2800296

[5] Vasan RS, Larson MG, Leip EP, Kannel WB, Levy D. Assessment of frequency of progression to hypertension in non-hypertensive participants in the Framingham Heart Study: a cohort study. Lancet. 2001;358:1682–6. 10.1016/S0140-6736(01)06710-1 11728544

[6] Tsujimoto T, Sairenchi T, Iso H, Irie F, Yamagishi K, Tanaka K, . Impact of obesity on incident hypertension independent of weight gain among nonhypertensive Japanese: the Ibaraki Prefectural Health Study (IPHS). J Hypertens. 2012;30:1122–8. 10.1097/HJH.0b013e328352b879 22487734

[7] Hu D, Hannah J, Gray RS, Jablonski KA, Henderson JA, Robbins DC, . Effects of obesity and body fat distribution on lipids and lipoproteins in nondiabetic American Indians: The Strong Heart Study. Obes Res. 2000;8:411–21. 10.1038/oby.2000.51 11011907

[8] Wang Y, Chen X, Song Y, Caballero B, Cheskin LJ. Association between obesity and kidney disease: a systematic review and meta-analysis. Kidney Int. 2008;73:19–33. 10.1038/sj.ki.5002586 17928825

[9] Gelber RP, Kurth T, Kausz AT, Manson JE, Buring JE, Levey AS, . Association between body mass index and CKD in apparently healthy men. Am J Kidney Dis. 2005;46:871–80. 10.1053/j.ajkd.2005.08.015 16253727

[10] Kramer H, Luke A, Bidani A, Cao G, Cooper R, McGee D. Obesity and prevalent and incident CKD: the Hypertension Detection and Follow-Up Program. Am J Kidney Dis. 2005;46:587–94. 10.1053/j.ajkd.2005.06.007 16183412

[11] Fox CS, Larson MG, Leip EP, Culleton B, Wilson PW, Levy D. Predictors of new-onset kidney disease in a community-based population. JAMA. 2004;291:844–50. 10.1001/jama.291.7.844 14970063

[12] Yamagata K, Ishida K, Sairenchi T, Takahashi H, Ohba S, Shiigai T, . Risk factors for chronic kidney disease in a community-based population: a 10-year follow-up study. Kidney Int. 2007;71:159–66. 10.1038/sj.ki.5002017 17136030

[13] Matsuo S, Imai E, Horio M, Yasuda Y, Tomita K, Nitta K, . Revised equations for estimated GFR from serum creatinine in Japan. Am J Kidney Dis. 2009;53:982–92. 10.1053/j.ajkd.2008.12.034 19339088

[14] Levey AS, Bosch JP, Lewis JB, Greene T, Rogers N, Roth D. A more accurate method to estimate glomerular filtration rate from serum creatinine: a new prediction equation. Modification of Diet in Renal Disease Study Group. Ann Intern Med. 1999;130:461–70. 10.7326/0003-4819-130-6-199903160-00002 10075613

[15] Levey AS, Eckardt KU, Tsukamoto Y, Levin A, Coresh J, Rossert J, . Definition and classification of chronic kidney disease: a position statement from Kidney Disease: Improving Global Outcomes (KDIGO). Kidney Int. 2005;67:2089–100. 10.1111/j.1523-1755.2005.00365.x 15882252

[16] Matsuzawa Y, Tokunaga K, Kotani K, Keno Y, Kobayashi T, Tarui S. Simple estimation of ideal body weight from body mass index with the lowest morbidity. Diabetes Res Clin Pract. 1990;10 Suppl 1:S159–64. 10.1016/0168-8227(90)90157-O 2286124

[17] Kambham N, Markowitz GS, Valeri AM, Lin J, D’Agati VD. Obesity-related glomerulopathy: an emerging epidemic. Kidney Int. 2001;59:1498–509. 10.1046/j.1523-1755.2001.0590041498.x 11260414

[18] Tozawa M, Iseki K, Iseki C, Oshiro S, Ikemiya Y, Takishita S. Influence of smoking and obesity on the development of proteinuria. Kidney Int. 2002;62:956–62. 10.1046/j.1523-1755.2002.00506.x 12164878

[19] de Jong PE, Verhave JC, Pinto-Sietsma SJ, Hillege HL; PREVEND study group. Obesity and target organ damage: the kidney. Int J Obes Relat Metab Disord. 2002;26 Suppl 4:S21–4. 10.1038/sj.ijo.0802213 12457295

[20] Wu Y, Liu Z, Xiang Z, Zeng C, Chen Z, Ma X, . Obesity-related glomerulopathy: insights from gene expression profiles of the glomeruli derived from renal biopsy samples. Endocrinology. 2006;147:44–50. 10.1210/en.2005-0641 16210374

[21] Kalupahana NS, Moustaid-Moussa N. The renin-angiotensin system: a link between obesity, inflammation and insulin resistance. Obes Rev. 2012;13:136–49. 10.1111/j.1467-789X.2011.00942.x 22034852

[22] Aneja A, El-Atat F, McFarlane SI, Sowers JR. Hypertension and obesity. Recent Prog Horm Res. 2004;59:169–205. 10.1210/rp.59.1.169 14749502

[23] Nickenig G, Bäumer AT, Grohè C, Kahlert S, Strehlow K, Rosenkranz S, . Estrogen modulates AT1 receptor gene expression in vitro and in vivo. Circulation. 1998;97:2197–201. 10.1161/01.CIR.97.22.2197 9631868

[24] Gallagher PE, Li P, Lenhart JR, Chappell MC, Brosnihan KB. Estrogen regulation of angiotensin-converting enzyme mRNA. Hypertension. 1999;33:323–8. 10.1161/01.HYP.33.1.323 9931124

[25] Dubey RK, Oparil S, Imthurn B, Jackson EK. Sex hormones and hypertension. Cardiovasc Res. 2002;53:688–708. 10.1016/S0008-6363(01)00527-2 11861040

[26] Nakamura M, Sato S, Shimamoto T. Improvement in Japanese clinical laboratory measurements of total cholesterol and HDL-cholesterol by the US Cholesterol Reference Method Laboratory Network. J Atheroscler Thromb. 2003;10:145–53. 10.5551/jat.10.145 14564083

