# Supplementary material for: The Dose-Response Relationship Between Body Mass Index and the Risk of Incident Stage ≥3 Chronic Kidney Disease in a General Japanese Population: The Ibaraki Prefectural Health Study (IPHS)
Source: J Epidemiol. 2014 Nov 5;24(6):444–51. doi: 10.2188/jea.JE20140028 (PMC4213218; doi:10.2188/jea.JE20140028)
Supplement: Abstract in Japanese. [file je-24-444-s001.pdf]

日本人におけるステージ 3 以上の慢性腎臓病発症リスクに対する body mass index の量・反応関係：茨城県健康研究

辻本　健彦[1,2]、西連地　利己[2,3]、磯　博康[4]、入江　ふじこ[5]、山岸　良匡[2,6]、渡辺宏[7]、田中　喜代次[1,2]、武藤　孝司[3]、大田　仁史[2]

１）筑波大学体育系
２）茨城県立健康プラザ
３）獨協医科大学医学部公衆衛生学講座
４）大阪大学大学院医学系研究科社会医学専攻公衆衛生学
５）茨城県保健福祉部保健予防課
６）筑波大学医学医療系社会健康医学
７）茨城県総合健診協会

【目的】日本人における body mass index（BMI）とステージ 3 以上の慢性腎臓病発症リスクとの関連を検討すること。
【方法】対象者は、茨城県内における 1993 年の基本健康診査の受診者で慢性腎臓病の既往歴のない 40～74 歳の男女 105611 人であった。対象者の慢性腎臓病発症を 2006 年まで追跡した。ステージ 3 以上の慢性腎臓病発症の定義は推定糸球体濾過量が 2 回以上の健診で 60 ml/min/1.73 m^2 未満となった時点、もしくは腎臓病治療開始時点とした。 BMI 区分（18.5 未満、18.5～20.9、21.0～22.9、23.0～24.9、25.0～26.9、27.0～29.9、30.0 以上）の慢性腎臓病発症に対する多変量調整ハザード比は Cox の比例ハザードモデルを用いて算出し、可能性のある交絡要因で調整した。
【結果】平均 5 年の追跡期間中に 19384 人（男性 6283 人、女性 14015 人）のステージ 3 以上の慢性腎臓病発症が確認された。ステージ 3 以上の慢性腎臓病発症に対する多変量調整ハザード比は、BMI が 21.0～22.9 を基準とした場合、男性で BMI が 23.0 以上、女性で BMI が 27.0 以上で有意に高値を示した。また、男女ともに、BMI とステージ 3 以上の慢性腎臓病発症率との間に量・反応関係が認められた。
【まとめ】肥満は、男女ともにステージ3以上の慢性腎臓病の発症リスクと関連する。

【キーワード】慢性腎臓病、body mass index、肥満、量・反応関係、疫学
